# Cannabis containing equivalent concentrations of delta-9-tetrahydrocannabinol (THC) and cannabidiol (CBD) induces less state anxiety than THC-dominant cannabis

**DOI:** 10.1007/s00213-022-06248-9

**Published:** 2022-10-13

**Authors:** Nadia R. P. W. Hutten, T. R. Arkell, F. Vinckenbosch, J. Schepers, R. C. Kevin, E. L. Theunissen, K. P. C. Kuypers, I. S. McGregor, J. G. Ramaekers

**Affiliations:** 1 Department of Neuropsychology & Psychopharmacology, Faculty of Psychology & Neuroscience, Maastricht University, Maastricht, Netherlands; 2 Centre for Human Psychopharmacology, Swinburne University of Technology, Melbourne, VIC Australia; 3 Department of Methodology & Statistics, Faculty of Psychology & Neuroscience, Maastricht University, Maastricht, Netherlands; 4 School of Psychology, Faculty of Science, The University of Sydney, Sydney, NSW Australia

**Keywords:** Delta-9-tetrahydrocannabinol, Cannabidiol, Anxiety, Healthy

## Abstract

**Rationale:**

*Delta*-9-tetrahydrocannabinol (THC), an active component of cannabis, can cause anxiety in some users during intoxication. Cannabidiol (CBD), another constituent of cannabis, has anxiolytic properties suggesting that cannabis products containing CBD in addition to THC may produce less anxiety than THC-only products. Findings to date around this issue have been inconclusive and could conceivably depend on moderating factors such as baseline anxiety levels in users.

**Objective:**

The present study examined whether anxiety following single doses of vaporised THC, CBD and THC/CBD might be explained by state and trait anxiety levels at baseline.

**Methods:**

A placebo-controlled, randomised, within-subjects study including 26 healthy recreational cannabis users tested the effects of vaporised THC-dominant cannabis (13.75 mg THC), CBD-dominant cannabis (13.75 mg CBD), THC/CBD-equivalent cannabis (13.75 mg THC/13.75 mg CBD) and placebo cannabis on anxiety. Self-rated trait anxiety was assessed with the State-Trait Anxiety Inventory (STAI). State levels of anxiety were objectively assessed with a computer-based emotional Stroop task (EST) and subjectively rated with the STAI-state questionnaire and a visual analogue scale.

**Results:**

Both THC and THC/CBD significantly increased self-rated state anxiety compared to placebo. State anxiety after THC/CBD was significantly lower than after THC alone. THC-induced anxiety was independent of anxiety at baseline. When baseline anxiety was low, CBD completely counteracted THC-induced anxiety; however, when baseline anxiety was high, CBD did not counteract THC-induced anxiety. There were no effects of any treatment condition on the EST.

**Conclusion:**

Overall, the study demonstrated that the THC/CBD-equivalent cannabis induces less state anxiety than THC-dominant cannabis.

**Supplementary Information:**

The online version contains supplementary material available at 10.1007/s00213-022-06248-9.

## Introduction

With the growing trend to legalise or decriminalise recreational and medical use of cannabis, the prevalence of cannabis consumption is expected to increase (Hall and Lynskey 2016; Han et al. 2018). The potency of recreational cannabis products has risen substantially in Europe and the USA over the past decade, as shown by higher levels of their psychoactive substance delta-9-tetrahydrocannabinol (THC) (Chandra et al. 2019). THC is mainly used recreationally to induce a subjective feeling of high (Curran et al. 2002), but it may also produce undesired feelings such as anxiety (Arkell et al. 2019; Bhattacharyya et al. 2010; D'Souza et al. 2004; Hunault et al. 2014; Karschner et al. 2011; Zuardi et al. 1982). Also, medical formulations of THC have been associated with anxiety in patients suffering from HIV wasting disease (Inc. 2017).

The anxiolytic properties of the non-intoxicating cannabis compound, cannabidiol (CBD), have shown to be promising in reducing symptoms in social anxiety disorder, generalised anxiety disorder, panic disorder and post-traumatic stress disorder in preclinical and clinical studies (Berger et al. 2020; Blessing et al. 2015; Masataka 2019; Skelley et al. 2020). Preclinical studies suggest that CBD has anxiolytic effects under high-stress conditions (i.e. foot shock prior to a light–dark emergence test) but not under low-stress conditions (i.e. light–dark emergence test or open field test) (Rock et al. 2017; Todd and Arnold 2016). Human studies have also indicated anxiolytic properties of CBD and reported reductions in stress-induced anxiety in healthy volunteers (de Souza Crippa et al. 2004; Linares et al. 2018; Zuardi et al. 2017b; Zuardi et al. 1993) and patients with Parkinson’s disease (de Faria et al. 2020).

Nabiximols, a plant-based medication containing THC and CBD in a ratio of 1:1, ranging from 2.7 mg THC/2.5 mg CBD to 32.4 mg THC/30 mg CBD oromucosal, is currently prescribed to relieve symptoms of multiple sclerosis and cancer-related pain (Krcevski‐Skvarc et al. 2018). A THC/CBD ratio of 1:1 is believed to provide the best balance between therapeutic effects and adverse effects, such as anxiety (Robson 2014). However, some patients using nabiximols still experienced THC-induced anxiety despite the presence of CBD in their formulation (Barnes 2006; Syed et al. 2014). Notably, the anxiolytic effects of CBD tend to occur at a much higher dose (300 mg orally) (Masataka 2019; Zuardi et al. 2017b) than would be delivered with therapeutic doses of nabiximols (e.g. 2.5–30 mg oromucosal) (GW Pharma Ltd. 2022). Also, clinical studies in healthy volunteers have reported only a minimal impact of CBD on THC-induced anxiety with THC to CBD ratios of 1:1 and 1:2 when vaporised (13.75 mg THC/13.75 CBD), consumed orally (0.5 mg/kg THC/1.0 mg/kg CBD) or after oromucosal use (5.4 mg THC/5.0 mg CBD and 16.2 mg THC/15.0 mg CBD) (Arkell et al. 2019; Zuardi et al. 1982; Karschner et al. 2011). These findings might indicate that other factors play a role in the anxiogenic or anxiolytic effects of THC/CBD.

Potential factors moderating CBD and THC effects on anxiety include both state and trait anxiety prior to cannabis intake. In preclinical studies, high levels of state anxiety, induced by the presence of an explicit stressor, may determine the anxiolytic effects of CBD (Rock et al. 2017). However, it is unknown whether baseline state anxiety also affects THC and THC/CBD-induced anxiety in humans. In addition, high trait anxiety is positively associated with increased selective attention towards anxiety-related stimuli, i.e. words or pictures (Mathews et al. 1997). This increased bias towards anxiety-related stimuli might also play a role in the increased state anxiety after THC inhalation. In line with this, those who score high on trait anxiety might experience greater relief of anxiety symptoms when treated with CBD.

Furthermore, while CBD appears only to produce anxiolytic effects in individuals with high states of anxiety (Freeman et al. 2019; Szkudlarek et al. 2019), CBD might only counteract high levels of THC-induced anxiety. Therefore, it is interesting to explore whether those who experience heightened anxiety after THC inhalation display lower anxiety levels after combined inhalation of THC and CBD.

The present study contain secondary, exploratory analyses of the anxiety data collected presented in a randomised controlled trial investigating THC and CBD effects on cognition and driving performance (Arkell et al. 2020). The current study compared the effects of inhaled CBD, THC and THC/CBD on anxiety (presented in Arkell et al. 2020) and further examined whether these effects depend upon moderating factors such as baseline state and trait anxiety levels. Furthermore, we aimed to explore whether individuals who experience high THC-induced anxiety levels display a more substantial reduction in anxiety symptoms when CBD is co-administered.

## Methods

### Design

This study involved a double-blind, placebo-controlled, within-subjects design with four treatment conditions separated by a minimum washout period of 7 days to avoid potential carry-over effects.

Treatment conditions were THC-dominant cannabis (13.75 mg THC), CBD-dominant cannabis (13.75 mg CBD), THC/CBD-equivalent cannabis (13.75 mg THC/13.75 mg CBD) and cannabis placebo. THC-dominant (THC 22% and CBD < 1%), CBD-dominant (THC < 1% and CBD 9%) and placebo (< 0.2% total cannabinoid content) cannabis varieties (Bedrocan) were used to deliver these target doses. The cannabis flos was delivered by the Office for Medicinal Cannabis. The order of treatment conditions was randomised across participants. Cannabis and placebo cannabis were self-administered by vaporisation at 200 ˚C (Mighty Medic, Storz & Bickel, Tuttlingen, Germany). Participants were instructed to inhale for 5 s, hold their breath for 3 s, exhale and repeat this until the vapour was no longer visible, leaving 30 s in between.

### Participants

Participants were 26 healthy occasional cannabis users (10 males; 16 females), aged 23.1 years on average (*SD* = 2.60). The frequency of cannabis use in the three months prior to study entrance was 10.50 times (*SD* = 13.57). Participants reported to have experience with other substances, including ecstasy (*N* = 7), amphetamines (*N* = 1), cocaine (*N* = 4), psilocybin (*N* = 7), LSD (*N* = 3) and ephedra (*N* = 1).

All assessments were conducted in English; however, not all participants in this study were native English speakers. The nationality of the participants was German (*N* = 13), Dutch (*N* = 3), Italian (*N* = 2), Slovenian (*N* = 2), British (*N* = 1), Finish (*N* = 1), Malaysian (*N* = 1), Portuguese (*N* = 1), Serbian (*N* = 1) and Sri Lankan (*N* = 1).

### Procedures

Participants were recruited through advertisements in university buildings in Maastricht, via social media and by word of mouth. Before inclusion, participants underwent a medical screening by a physician. Their general health was checked, and blood and urine samples were taken for standard blood chemistry, haematology and urinalysis.

Inclusion criteria were written informed consent; age 20–50 years; occasional cannabis use (defined as; > 10 lifetime exposures and < 2 per week in the last 12 months); good physical health as determined by medical history and medical examination; absence of any major medical, endocrine and neurological conditions; and BMI between 20 and 28 kg/m^2^. Exclusion criteria were history of drug abuse or addiction; current or history of psychiatric disorder, including anxiety-related disorders; cardiovascular abnormalities; hypertension; liver dysfunction; any serious prior adverse response to cannabis; and pregnancy or lactation.

Prior to the test days, participants visited the test facilities for a training session to familiarise them with the tests and test procedures. Participants were trained to understand and perform the tasks optimally. They filled out the trait section of the State-Trait Anxiety Inventory questionnaire (STAI-trait). Participants were instructed to abstain from illicit substance use and alcoholic beverages, respectively, 7 days and 1 day before each test day. Instructions for the test days were to have a light breakfast at home, to consume their regular amount of caffeine and to arrive well rested at the experimental facilities at 9 AM. They were screened for alcohol in breath and drugs of abuse in saliva (cocaine, opiates, benzodiazepine, methamphetamine, amphetamine, MDMA and THC), and women were tested for pregnancy in urine.

When tests were negative, baseline state anxiety was measured by a visual analogue scale (VAS), a peripheral venous catheter was inserted, and a blood sample was taken. The treatment was vaporised at 9.45 AM. After inhalation, an emotional Stroop task (EST) was conducted, and participants rated their state anxiety on the state section of the STAI (STAI-state) and VAS. The VAS was assessed repeatedly up to 5.5 h after inhalation, and blood samples were taken at similar intervals (Table 1).

**Table 1:** Overview of measurements taken during the training session and test days

		Test days						
		Time relative to administration (minutes)						
Measurements	Training session	Baseline	0	25	130	200	240	320
STAI-trait	X							
STAI-state			X					
VAS		X	X	X	X	X	X	
Emotional Stroop			X					
Blood sample		X	X	X	X	X		X

*STAI*, State-Trait Anxiety Inventory; *VAS*, visual analogue scale

The study was conducted according to the code of ethics on human experimentation established by the declaration of Helsinki amended in Fortaleza (World Medical Association, 2013) and it was approved by the Medical Ethics Committee of the Academic Hospital of Maastricht and Maastricht University. A permit for obtaining, storing and administering cannabis was obtained from the Dutch Drug Enforcement Administration. Participants were reimbursed for their invested time.

### State-Trait Anxiety Inventory (STAI)

The State-Trait Anxiety Inventory was used to measure self-rated anxiety. It consists of a trait and a state anxiety section (Spielberger 1983a), assessing respectively relatively stable aspects of anxiety proneness and the current anxiety level. Both sections consist of 20 four-point Likert statements with answer options ranging from “almost never” to “almost always” for the trait section and “not at all” to “very much so” for the state section. The scores of both scales have a min–max score range of 20–80, with a higher score indicating higher anxiety. Scores on the STAI-trait were used as a moderator variable and the scores on the STAI-state as a dependent variable. State anxiety scores higher than 39–40 have been suggested to indicate clinically significant symptoms (Julian 2011). Normative state and trait anxiety scores for healthy adults and college students range, respectively, between 36–38 and 38–40 (Spielberger 1983b).

### Visual analogue scale (VAS)

Participants were asked to indicate their level of anxiety throughout the day on a 10-cm-long horizontal VAS scale with zero meaning “not anxious” and ten “very anxious”. Data of the VAS measured prior to treatment inhalation was used as a moderator variable, and the VAS scores after inhalation were used as dependent variables.

### Emotional Stroop task (EST)

A computerised emotional Stroop task (EST) was used to assess implicit anxiety. Twenty anxiety-related and twenty anxiety-neutral words (Richards et al. 1992) were presented in a coloured font (blue, red, green or yellow) on a grey background. Each word was presented twice in random order resulting in a total of 80 trials. A trial started with a fixation cross, presented for 100 ms in the centre of the screen, followed by the word, which remained visible until a response was made. Participants were instructed to identify the word colour by pressing the correct button on the response box as fast as possible and to ignore the word content.

Prior to these sessions, participants were familiarised with the task procedure during the training session by presenting 40 neutral words twice (80 trials). All word lists used are shown in the Supplement (Table S1).

The primary outcome variable of the EST was calculated by subtracting the reaction time for the neutral words from the reaction time of the anxiety-related words, with a positive score indicating increased attentional bias to anxiety-related words. In addition, difference scores were calculated for the number of correct responses between anxiety-related and neutral words as a control measure, with a negative score, meaning more correct responses to neutral words and thereby avoiding anxiety-related stimuli, hence indicating anxious behaviour.

### Pharmacokinetics

Blood samples were taken at baseline and at 0 (directly), 25, 130, 200 and 320 min after treatment inhalation to determine THC, 11-OH-THC, 11-THC-COOH, CBD and 7-OH-CBD and 7-COOH-CBD concentrations. Blood samples were centrifuged, and plasma was extracted and stored at − 20ºC until analysis. Plasma was analysed via liquid chromatography-tandem mass spectrometry (LC–MS/MS) (Kevin et al. 2017; Schwope et al. 2011).

### Statistical analyses

Data were analysed by means of the statistical package IBM SPSS Statistics (version 25).

All data were analysed with linear mixed models (LMMs) with restricted maximum likelihood method (REML). In all analyses, compound symmetry was specified as covariance structure for the repeated factor treatment (4 levels). In the analysis of VAS, an unstructured covariance structure was further specified for the repeated factor time (5 levels).

Trait anxiety (STAI-trait) was added as a moderator variable and a moderator by treatment interaction in all models (STAI-state, VAS and EST) to examine whether trait anxiety moderated treatment effects. Baseline state anxiety was added as a moderator variable and as a moderator by treatment interaction to the statistical model of the VAS to examine whether baseline state anxiety moderated treatment effects. Note, no baseline anxiety states were recorded with the STAI-state and EST. When a moderator by treatment interaction was statistically non-significant, it was removed from the LMM; when subsequently a moderator main effect was statistically non-significant, it was also removed from the model. In case of a significant main treatment effect, planned pairwise comparisons were performed. In case of a significant treatment by time interaction, pairwise comparisons were performed between treatment conditions at each level of time. In the case of a treatment by moderator interaction, separate planned pairwise comparisons were performed to examine the effects of treatment on low (i.e. mean – 1SD), medium (i.e. mean) and high (i.e. mean + 1SD) moderator values, respectively.

Because no parallel versions of the emotional Stroop task were used, the data were analysed to examine potential habituation effects using LMMs, including the fixed factors treatment (4 levels), test day (4 levels) and treatment by test day interaction.

To explore whether individuals who experience high THC-induced anxiety levels display a more substantial reduction in anxiety symptoms when CBD is co-administered, STAI-state and the VAS (peak) difference scores between THC condition and placebo (THC – placebo) were correlated with the difference scores between THC/CBD and THC (THC/CBD – THC). Since baseline state anxiety played a role in the analyses of state anxiety measured by the VAS, data of the VAS was baseline-corrected in this correlation analysis. To account for possible regression to the mean artefacts, additional tests of homogeneity of variances were conducted to assess whether a significant difference of variance was present between THC condition and THC/CBD condition. In addition, binomial tests were employed to compare the proportion of participants (%) that showed a reduction (1) versus an increase (0) in treatment-induced state anxiety measured by the STAI-state and VAS. For the VAS, peak difference scores were used. The test proportion was set at 50%.

To examine the association of blood plasma concentrations with treatment-induced anxiety, difference scores of the STAI-state, VAS (peak) scores and EST data from placebo (drug–placebo) were correlated with the plasma cannabinoid concentrations of the same time points when the anxiety measures were assessed. The VAS peak scores for the THC condition were recorded directly after inhalation, and the peak anxiety increase after THC/CBD inhalation was recorded at 25 min after inhalation. Since baseline state anxiety played a role in the analyses of state anxiety measured by the VAS, data of the VAS was baseline-corrected in this correlation analysis.

The alpha criterion level of statistical significance for all analyses was set at *p* = 0.05. Pairwise comparisons were Bonferroni-corrected by multiplying the *p* values by 4, the total number of predefined comparisons: THC vs placebo, THC/CBD vs placebo, CBD vs placebo and THC vs THC/CBD. Correlation analyses including blood plasma and induced anxiety were Bonferroni-corrected by multiplying the *p* value by 3, total number of active treatment groups (CBD, THC and THC/CBD). Pearson correlations were used in normally distributed data and Kendall’s Tau-b in non-normally distributed data.

## Results

### STAI-trait anxiety

Overall, participants had a mean score of 33.13 (SD = 7.85) on *trait* anxiety.

### STAI-state anxiety

**Fig. 1:**
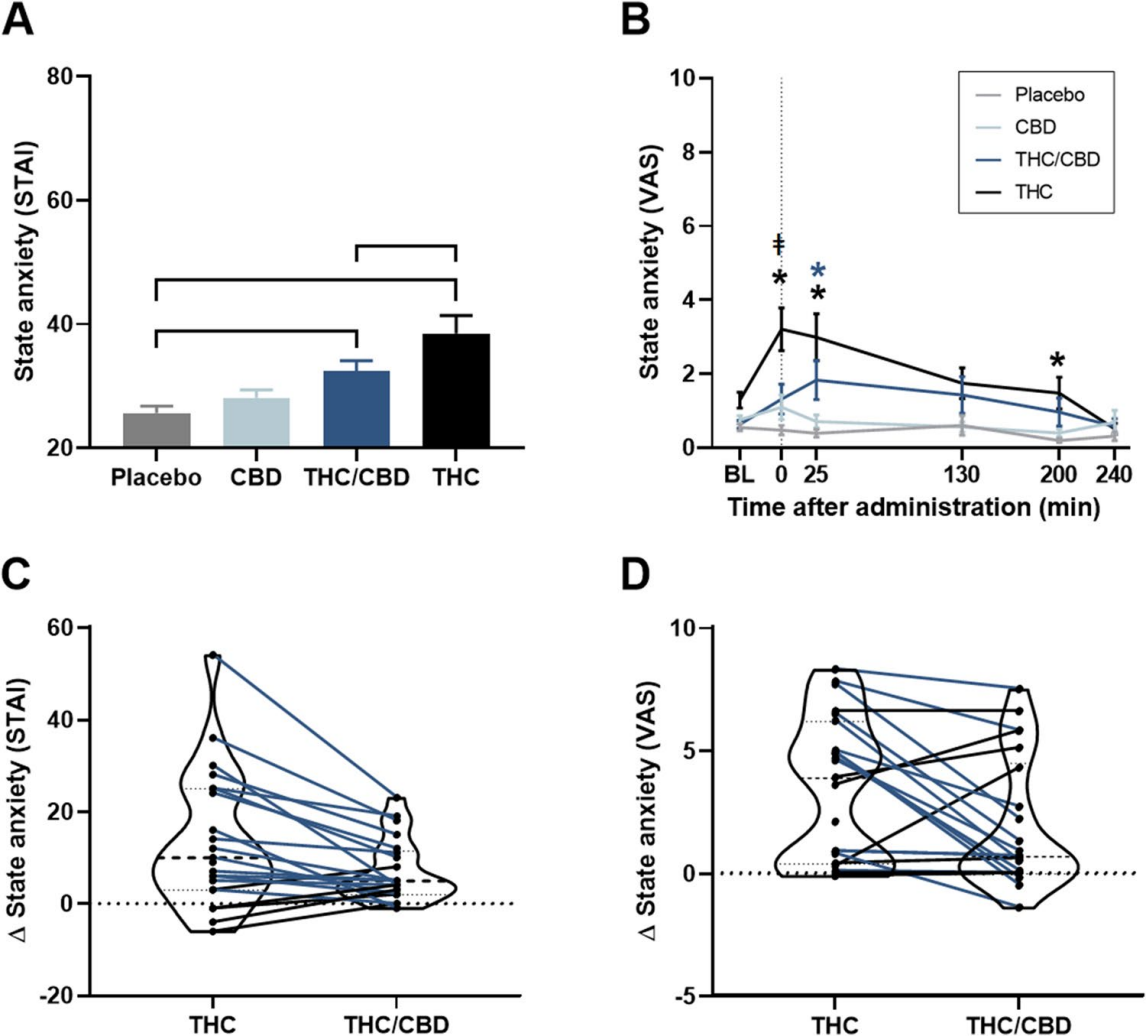
Mean (SE) state anxiety (STAI) per treatment (**A**); mean (SE) state anxiety (VAS) per treatment over time (**B**); violin plots of drug–placebo differences scores on state anxiety (STAI) (**C**); and drug–placebo difference peak scores on state anxiety (VAS) (**D**) with connected individual data points showing less (blue lines) or more (black lines) anxiety after THC/CBD compared to THC. ┌┐ significant treatment contrasts, * significant difference between the treatment condition and placebo and ǂ significant difference between THC and THC/CBD condition (*p* = 0.02). STAI, State-Trait Anxiety Inventory; VAS, visual analogue scale; BL, baseline

LMM did not reveal significant moderator effects of trait anxiety and trait anxiety by treatment interaction and therefore were not included in the model. There was a significant main treatment effect (*F*_*3,67.51*_ = 17.11, *p* < 0.01); subsequent contrasts revealed that participants felt more anxious in the THC (*p* < 0.01) and THC/CBD (*p* < 0.01) conditions compared to placebo; the THC/CBD-induced anxiety was significantly less compared to THC-induced anxiety (*p* = 0.01) (Fig. 1A and Table S3). There was no significant difference in anxiety between CBD and placebo (Table S3).

Pearson correlation analysis revealed that those who experienced greater anxiety in the THC condition showed a larger decrease in anxiety when CBD was co-administered (*r*(21) =  − 0.92, *p* < 0.01). Homogeneity test of variances showed no significant difference in the variance between the THC/CBD and the THC condition (*F*(1,40) = 2.74, *p* = 0.11); therefore, it can be concluded that the current correlation analyses are not driven by an artefact of regression to the mean. Figure 1C shows violin plots of THC- and THC/CBD-induced state anxiety with connected individual data points showing less or more state anxiety after THC/CBD compared to THC. Binomial test indicated that the majority of participants (81%) showed a reduction in state anxiety following THC/CBD administration compared to THC (*p* < 0.01).

### VAS state anxiety

LMMs revealed significant effects of treatment, time, treatment by time interaction and the moderators baseline state anxiety, baseline state anxiety by treatment and trait anxiety by treatment interactions (all *p* < 0.05) (Table S2). There was no main effect of the moderator trait anxiety. Pairwise comparisons between treatment conditions (averaged across time points) and at each level of time are presented in Fig. 1C and Table S3. Compared to placebo, THC significantly induced anxiety immediately (time 0), 25 and 200 min after inhalation, while THC/CBD only significantly increased anxiety at 25 min after inhalation. The THC/CBD-induced anxiety was significantly less compared to THC-induced anxiety directly after inhalation.

**Fig. 2:**
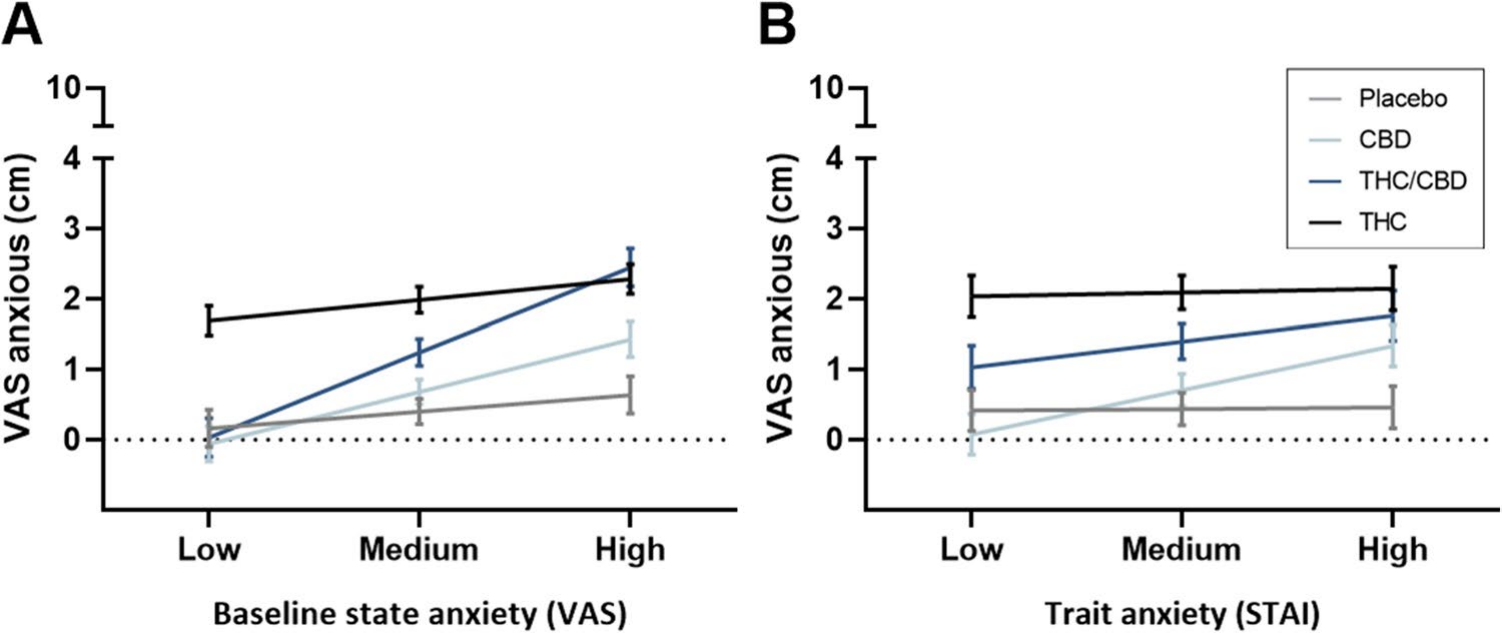
Mean (SE) state anxiety (VAS) per treatment at each level (low, medium and high) of baseline state (**A**) and trait (**B**) anxiety. VAS, visual analogue scale; STAI, State-Trait Anxiety Inventory

Separate treatment contrasts for low, medium and high moderator values are shown in Fig. 2 and Table 2. The mean differences of THC and placebo on the VAS and the corresponding 95%CI are approximately equal across the different values of the moderators, indicating that THC-enhanced anxiety compared to placebo is independent of baseline and trait anxiety. There was no evidence of an increase in anxiety following THC/CBD inhalation when baseline or trait anxiety was low, but increased anxiety was experienced when baseline or trait anxiety was medium to high. CBD did not counteract THC-induced anxiety when baseline anxiety was high, partly counteracted THC-induced anxiety when baseline anxiety was medium and counteracted THC-induced anxiety completely when baseline anxiety was low. CBD only counteracted THC-induced anxiety when trait anxiety was low. There was no evidence that CBD affects anxiety compared to placebo at all three values of baseline and trait anxiety.

**Table 2:** Bonferroni-corrected pairwise comparisons between treatment conditions on mean state anxiety (VAS) at each level of baseline state and trait anxiety (low, medium and high)

Baseline state anxiety									
	Low			Medium			High		
Mean VAS	Mean difference	*p*	95% CI	Mean difference	*p*	95% CI	Mean difference	*p*	95% CI
CBD-PLA	− 0.22	> 0.99	− 0.92, 0.48	0.28	> 0.99	− 0.20, 0.77	0.79	0.11	0.09, 1.49
THC/CBD-PLA	− 0.13	> 0.99	− 0.86, 0.60	0.84	<0.01*	0.34, 1.34	1.81	<0.01*	1.08, 2.55
THC-PLA	1.53	<0.01*	0.88, 2.18	1.59	<0.01*	1.09, 2.09	1.65	<0.01*	0.99, 2.31
THC-THC/CBD	1.66	<0.01*	0.99, 2.33	0.75	0.02*	0.24, 1.26	− 0.17	> 0.99	− 0.83, 0.50
Trait anxiety									
	Low			Medium			High		
Mean VAS	Mean difference	*p*	95% CI	Mean difference	*p*	95% CI	Mean difference	*p*	95% CI
CBD-PLA	− 0.34	> 0.99	− 1.03, 0.36	0.27	> 0.99	− 0.30, 0.84	0.88	0.06	0.17, 1.58
THC/CBD-PLA	0.62	0.35	− 0.09, 1.33	0.96	<0.01*	0.36, 1.56	1.30	<0.01*	0.50, 2.10
THC-PLA	1.63	<0.01*	0.93, 2.32	1.66	<0.01*	1.07, 2.24	1.69	<0.01*	0.96, 2.42
THC-THC/CBD	1.01	0.02*	0.29, 1.72	0.70	0.10	0.89, 1.31	0.39	> 0.99	− 0.43, 1.20

*VAS*, visual analogue scale; *CBD*, cannabidiol; *THC*, delta-9-tetrahydrocannabinol; *PLA*, placebo

* indicates significant difference after Bonferroni correction

Kendall Tau-b correlation analyses revealed that those who experienced greater THC-induced state anxiety showed a greater decrease in state anxiety when CBD was co-administered (*r*(22) =  − 0.81, *p* < 0.01). Homogeneity test of variances showed no significant difference in the variance between the THC/CBD and the THC condition (*F*(1,42) = 0.48, *p* = 0.49), therefore it can be concluded that the current correlation analyses are not driven by an artefact of regression to the mean. Figure 1D shows violin plots of THC and THC/CBD-induced state anxiety with connected individual data points showing less or more state anxiety after THC/CBD compared to THC. Binomial test, however, revealed no significant difference in the proportion of participants with decreased (55%) or increased (45%) anxiety following THC/CBD as compared to THC (*p* = 0.83).

### Emotional Stroop

There was no habituation effect across test days, and there was no significant main treatment effect (Table S4) or trait and treatment by trait interaction.

### Blood plasma and anxiety measures

Statistical analyses and details of blood plasma concentrations for all time points can be found in Arkell et al. (2020). The maximum blood plasma concentrations (SD) of THC, 11-OH-THC, CBD and 7-OH-CBD for all treatment conditions are presented in Table S5.

**Fig. 3:**
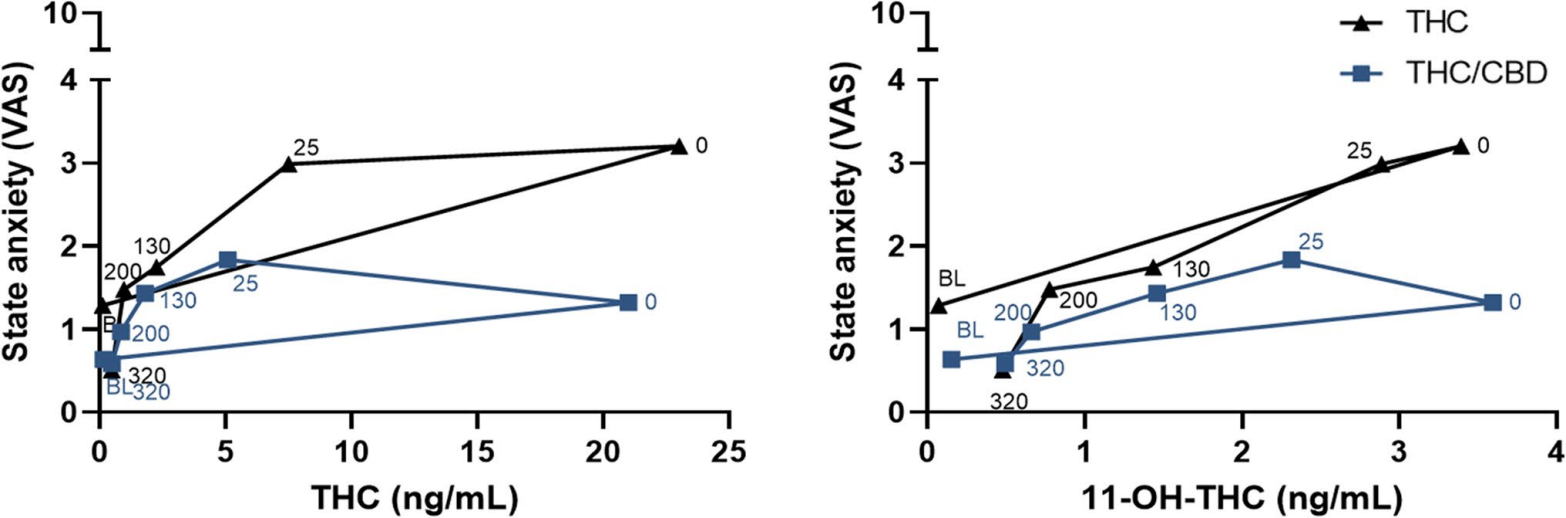
Mean state anxiety scores on the VAS plotted against the average of THC and 11-OH-THC plasma concentrations over time, for the THC and THC/CBD condition. VAS, visual analogue scale; BL, baseline

Correlation analyses revealed that increased state anxiety measured with the STAI correlated positively with maximum plasma concentrations of THC (*p* = 0.05) and 11-OH-THC (*p* < 0.01) after the THC but not after THC/CBD inhalation. All other correlation analyses were non-significant (Table S6). Figure 3 presents counterclockwise hysteresis relationships between state anxiety measured by the VAS and blood plasma concentrations of THC and 11-OH-THC for the THC and THC/CBD conditions.

## Discussion

The present study aimed to examine CBD, THC and THC/CBD effects on state anxiety and whether baseline state and trait anxiety levels moderate these effects. A secondary aim was to explore whether participants who experience heightened anxiety following THC inhalation experience a stronger relief of anxiety symptoms when THC is given in combination with CBD. THC and THC/CBD both increased self-rated state anxiety compared to placebo. Directly after inhalation, self-rated state anxiety after THC/CBD was significantly lower compared to THC. CBD, by itself, did not significantly change anxiety ratings on any of the anxiety measures. THC-induced anxiety was independent from baseline state and trait anxiety. CBD counteracted THC-induced anxiety completely when baseline anxiety was low, partly counteracted THC-induced anxiety when baseline anxiety was medium and did not counteract THC-induced anxiety when baseline anxiety was high. CBD only counteracted THC-induced anxiety when trait anxiety was low. No treatment effects were found on state anxiety when measured with the EST.

The present study showed that CBD partially blocked THC-induced anxiety when the two substances were delivered in equivalent concentrations, which is in line with previous studies (Arkell et al. 2019; Karniol et al. 1974). This might suggest that increasing the CBD-to-THC ratio would be even more effective in counteracting the THC-induced effects. However, preclinical studies showed that a higher CBD-to-THC ratio (5:1) did not augment the anxiogenic-like behaviour of THC, nor did they accentuate the THC effects compared to a ratio of 1:1 (Rock et al. 2017; Todd and Arnold 2016). While THC may have a bimodal effect on anxiety (with low doses being anxiolytic and high doses anxiogenic (Moreira et al. 2009; Rubino et al. 2008)) and the effects of CBD on anxiety may follow an inverted U-shape dose–response curve (Blessing et al. 2015), no clear THC/CBD dose–response relationship has been established yet (Niesink and van Laar 2013). Next to that, the THC/CBD interaction effects are also dependent on the order of administration and the route of administration (Zuardi et al. 2012).

Combined treatment of THC and CBD delayed the onset of state anxiety, reduced its magnitude and shortened its duration compared to inhalation of THC alone. A similar pattern with CBD on THC-induced hypothermia was shown in rodents (Todd and Arnold 2016). Possible underlying mechanisms of THC/CBD interactions are complex. THC can modulate anxiety by binding to the orthosteric sites as a partial agonist on the cannabinoid type 1 (CB1) receptors (Hayakawa et al. 2008; Laprairie et al. 2015; Zarrindast et al. 2008); it acts as a full agonist at CB1 receptors on GABA axon terminals and thereby inhibiting GABA release at high doses (Laaris et al. 2010; Lutz et al. 2015). CBD, on the other hand, is suggested to decrease THC-induced anxiety either by acting as a negative allosteric modulator of CB1 receptors (Hayakawa et al. 2008; Laprairie et al. 2015), or via inhibiting fatty acid amide hydrolase, thereby increasing anandamide concentrations which then compete with THC for CB1 receptor binding (Pertwee 2008), or by activating transient receptor potential vanilloid 1 which oppose the effects of CB1 receptors (Iannotti et al. 2014; Lisboa and Guimaraes 2012), or via modulation of 5-HT1a receptors (Zuardi et al. 2017a). Overall, these possible mechanisms might explain the reduction of THC-induced anxiety, but it does not explain how CBD is able to delay and shorten THC-induced anxiety.

Baseline state and trait anxiety moderated the effects of cannabis on anxiety ratings after cannabis inhalation in healthy volunteers. This moderation of baseline state and trait anxiety on anxiety experienced after cannabis inhalation may interest patients who use medicinal cannabis to relieve symptoms of multiple sclerosis or cancer-related pain or individuals who use cannabis recreationally. However, the finding that CBD only counteracted THC-induced anxiety when trait anxiety was low might indicate that patients with anxiety disorders or recreational users with a general tendency to be anxious might not benefit from the addition of CBD to THC. Alternatively, THC-induced increments in anxiety were relatively mild in the current study and only 1 subject achieved state anxiety levels of clinical relevance (state STAI > 40). Therefore, we cannot exclude that the magnitude of THC-induced anxiety as well as the anxiolytic effect of CBD might differ in anxious patients. Future studies should include anxiety patients or participants with a clinically significant trait anxiety score, to examine whether CBD will counteract THC-induced anxiety in these particular populations. In general, the more anxious a person is before inhalation, the less CBD attenuates THC-induced anxiety. Thus, if one experiences anxiety after using THC-only cannabis, it may be wise to use a combination THC/CBD cannabis while minimising anxiety before inhalation.

The present study found a strong negative correlation between THC-induced anxiety and the reduction in anxiety symptoms by CBD co-administration. This correlation indicates that for those who experienced high levels of THC-induced anxiety, CBD reduced the anxiety more strongly than those who only experienced a moderate increase in anxiety after THC administration. On the contrary, in participants who experienced a reduction or a small increase in state anxiety following THC, the addition of CBD increased their anxiety. A binominal test showed that most participants experienced a reduction in anxiety following THC/CBD compared to THC when measured with the STAI-state. However, the results of the STAI-state need to be interpreted with caution. The STAI-state was only assessed directly after inhalation when THC-induced anxiety had not reached its maximum effect in the THC/CBD condition. The VAS measured state anxiety repeatedly over time, thereby demonstrating the delayed onset of THC/CBD-induced anxiety and showing that about half of the participants experienced a lower peak state of anxiety following THC/CBD-equivalent cannabis than THC-dominant cannabis. Since the VAS was corrected for baseline state anxiety, other individual factors such as genetic makeup linked to CB1 receptor density (Witkin et al. 2005) may explain why only half of the participants experienced decreased anxiety following THC/CBD-equivalent compared to THC-dominant cannabis.

THC-induced state anxiety (STAI scores) moderately correlated with THC and 11-OH-THC blood plasma concentrations but not after THC/CBD inhalation. Therefore, THC and 11-OH-THC in blood plasma was not a good predictor of state anxiety when CBD is co-administered. In line with this, counterclockwise hysteresis loops show that the blood plasma concentration of both THC and 11-OH-THC were equal in the THC and THC/CBD conditions, while state anxiety (VAS scores) differed. These findings indicate that CBD does not alter THC concentrations in blood plasma.

Cannabis did not affect attentional bias towards anxiety-related stimuli, not even in the THC condition. A previous study by Richards et al. (1992) only found an attentional bias towards anxiety-related stimuli on the emotional Stroop task in a group scoring high on trait and state anxiety but not in a group scoring low on this trait and state. However, the score on state anxiety in the present study was lower than the state anxiety scores described in Richards et al. (1992), which induced an attentional bias towards anxiety-related stimuli (Mathews and Macleod 1985; Richards et al. 1992). The lack of THC-induced attentional bias towards anxiety-related stimuli on the EST might be due to the wide range of nationalities included, with the majority not having English as a mother tongue. Or because the present EST was set up using a mixed trial design by which the effect one of a negative item could affect the response to a following neutral item (sustained attentional bias), whereas using a block design, all items of a block are negative, and therefore, the effects on anxiety-related words are more pronounced (Ben-Haim et al. 2016). Future study needs to examine the effects of THC, CBD and THC/CBD by using a block design and a baseline measure of the emotional Stroop task.

The current study examined the acute effects of CBD on THC-induced anxiety in occasional cannabis users. These may differ from those in chronic cannabis users who may be at risk of higher levels of anxiety or the development of anxiety disorder (Patton et al. 2002; Xue et al. 2021). When repeatedly treated with THC-only cannabis (3 mg/kg), rodents showed an onset in anxiety over time; however, no onset in anxiety was seen when rodents were treated with THC/CBD (3 mg/kg THC/3 mg/kg CBD) (Murphy et al. 2017). This could indicate that CBD might also be able to prevent anxiety when THC is consumed chronically. However, the role of state and trait anxiety in the long-term use of cannabis is not known and should be further investigated.

This study was not without its limitations. This study was part of a larger trial, and therefore, only baseline state anxiety was measured using the VAS scale as it was easier to implement time-wise. Unfortunately, baseline state anxiety was not assessed with the STAI-state and EST. Future studies need to assess baseline state anxiety prior to treatment inhalation for every anxiety measurement used separately. In addition, lab-based experimental studies in humans are designed to make participants feel comfortable and at ease, which can be argued to not represent stressful real-life situations. As seen in previous preclinical studies, CBD only reduces states of anxiety when an explicit stressor is present (Rock et al. 2017). The current study had such a real-life stress situation, as participants needed to drive on the road after cannabis inhalation, which could provoke anxiety in some (Arkell et al. 2020). However, on-the-road driving tests occurred at 40 to 100 min and 240 to 300 min post-vaporisation (Arkell et al. 2020), and state anxiety was not measured during the driving test but 30 min before and 30 min after the driving test. Still, it cannot be ruled out that the significant difference in THC-induced anxiety compared to placebo at 200 min after inhalation could be due to anticipation of the participant of the second driving test. Nevertheless, future studies need to examine whether CBD is able to counteract THC-induced anxiety completely when an explicit stressor is present.

Another limitation of the current study is the use of a single dose of THC and CBD. The present findings might not be generalisable to higher doses of THC. Higher doses of THC could elicit higher states of anxiety. The mean score of state anxiety after THC inhalation in the present study was relatively low and below the level of clinical significance (state STAI < 40) (Julian 2011). Future studies should therefore include higher doses of THC and THC/CBD.

In conclusion, the present study showed that cannabis containing equivalent concentrations of THC and CBD induces less self-rated state anxiety compared to THC-only cannabis in healthy volunteers. Baseline state and trait anxiety moderated THC/CBD-induced anxiety but not THC-induced anxiety. The THC/CBD combination might be more favourable in clinical settings, and it may be a reasonable public health strategy to encourage cannabis breeds containing THC/CBD mixtures where recreational use of cannabis is now legal.

## Supplementary Information

Below is the link to the electronic supplementary material.

Supplementary file1 (DOCX 26 KB)
