# Supplementary material for: Cannabis containing equivalent concentrations of delta-9-tetrahydrocannabinol (THC) and cannabidiol (CBD) induces less state anxiety than THC-dominant cannabis
Source: Psychopharmacology (Berl). 2022 Oct 13;239(11):3731–41. doi: 10.1007/s00213-022-06248-9 (PMC9584997; doi:10.1007/s00213-022-06248-9)
Supplement: Supplementary file 1 — Supplementary file1 (DOCX 26 KB) [file 213_2022_6248_MOESM1_ESM.docx]

**Supplementary Material -** *Cannabis containing equivalent concentrations of delta-9-tetrahydrocannabinol (THC) and cannabidiol (CBD) induces less state anxiety than THC-dominant cannabis*

**Methods**

***Emotional Stroop task***

*Table S1.* Neutral words used for training session and anxiety-related words with anxiety matched neutral words used for test days.

| **Neutral Words (Training session)** | |
| --- | --- |
| Hawk | Ketchup |
| Cane | Coarse |
| Muddy | Custom |
| Truck | Trumpet |
| Lump | Radiator |
| Swamp | Highway |
| Boxer | Whistle |
| Trunk | Repentant |
| Alien | Privacy |
| Rattle | Scissors |
| Limber | Nursery |
| Mystic | Pamphlet |
| Salute | Nonsense |
| Clumsy | Appliance |
| Vanity | Sheltered |
| Spray | Skeptical |
| Invest | Sentiment |
| Icebox | Nonchalant |
| Insect | Thermometer |
| Hammer | Lighthouse |
| **Anxiety-related** | **Anxiety-Matched Neutral** |
| Weak | Take |
| Worried | Bramble |
| Agony | Verse |
| Panicky | Sections |
| Failure | Clothes |
| Nervous | Picture |
| Helpless | Interest |
| Terrified | Margarine |
| Painful | Around |
| Die | Cup |
| Sickness | Material |
| Disease | Library |
| Tragedy | Whistle |
| Accident | Instead |
| Suffering | Something |
| Cancer | Taller |
| Paralyzed | Expensive |
| Despair | Service |
| Distressed | Understand |
| Coffin | Lesser |

**Results**

*Table S2.* Linear Mixed Model of the VAS.

|  | *F (df)* | *p* |
| --- | --- | --- |
| Treatment | 5,15 (3, 60.77) | **<0.01** |
| Time | 7.61 (4, 87) | **<0.01** |
| Treatment x Time | 3.24 (12, 87) | **<0.01** |
| *Moderators* |  |  |
| VAS baseline | 36.70 (1, 51.36) | **<0.01** |
| VAS baseline x Treatment | 6.22 (3, 69.03) | **<0.01** |
| STAI-trait | 0.74 (1, 20.95) | 0.40 |
| STAI-trait x Treatment | 2.88 (3, 58.01) | **0.04** |

*Note:* VAS: Visual Analogue Scale; STAI: State-Trait Anxiety Inventory.

*Table S3.* Bonferroni-corrected pairwise comparisons between Treatment conditions (STAI-state and VAS) and at each level of Time (VAS).

|  | **CBD-PLA** | | **THC/CBD-PLA** | | **THC-PLA** | | **THC-THC/CBD** | |
| --- | --- | --- | --- | --- | --- | --- | --- | --- |
|  | *p* | 95% CI | *p* | 95% CI | *p* | 95% CI | *p* | 95% CI |
| **STAI-state** | 0.88 | 1.46, 6.18 | **<0.01** | 2.76, 10.89 | **<0.01** | 9.30, 17.20 | **0.01** | -10.54, -2.30 |
| **VAS** | >0.99 | -0.28, 0.75 | **<0.01** | 0.42, 1.49 | **<0.01** | 0.98, 2.04 | 0.18 | 0.01, 1.10 |
| **Time (min)** |  |  |  |  |  |  |  |  |
| **0** | 0.98 | -0.40, 1.55 | 0.24 | -0.04, 1.98 | **< 0.01** | 1.69, 3.69 | **<0.01** | 0.70, 2.76 |
| **25** | > 0.99 | -0.81, 1.27 | **0.02** | 0.51, 2.61 | **< 0.01** | 1.49, 3.61 | 0.33 | -0.13, 2.06 |
| **130** | > 0.99 | -0.99, 0.78 | 0.16 | 0.04, 1.87 | 0.10 | 0.14, 1.96 | > 0.99 | -0.84, 1.03 |
| **200** | > 0.99 | -0.60, 0.88 | 0.09 | 0.13, 1.66 | **0.01** | 0.44, 1.95 | > 0.99 | -0.48, 1.07 |
| **240** | 0.85 | -0.83, 0.19 | 0.70 | -0.17, 0.89 | > 0.99 | -0.46, 0.58 | > 0.99 | -0.84, 0.23 |

*Note:* STAI: State-Trait Anxiety Inventory; VAS: Visual Analogue Scale; CI: confidence interval.

*Table S4.* Final Linear Mixed Models of the Emotional Stroop without non-significant Trait moderator effects.

| **Habituation effect** | **Test day** | | **Test day x Treatment** | |
| --- | --- | --- | --- | --- |
|  | *F (df)* | *p* | *F (df)* | *p* |
| Number correct | 0.33 (3, 56.41) | 0.80 | 1.10 (9, 48.59) | 0.38 |
| Reaction time | 0.38 (3, 49.96) | 0.77 | 0.20 (9, 43.88) | 0.99 |
| **Treatment effect** | **Treatment** | |  | |
|  | *F (df)* | *p* |  |  |
| Number correct | 0.58 (3, 68.17) | 0.63 |  |  |
| Reaction time | 0.32 (3, 59.873) | 0.81 |  |  |

*Table S5.* Maximum blood plasma concentration (SD) of THC, 11-OH-THC, CBD and 7-OH-CBD (ng/mL) for all treatment conditions.

|  | **THC** (ng/mL) | **11-OH-THC** (ng/mL) | **CBD** (ng/mL) | **7-OH-CBD** (ng/mL) |
| --- | --- | --- | --- | --- |
| **Placebo** | 0.27 (0.55) | 0.18 (0.55) | 0.07 (0.34) | 0.08 (0.40) |
| **THC** | 22.91 (12.58) | 3.35 (2.17) | 0.02 (0.07) | <0.01 (<0.01) |
| **THC/CBD** | 19.98 (3.36) | 3.51 (3.46) | 13.92 (8.47) | 1.18 (1.28) |
| **CBD** | 1.75 (2.13) | 0.85 (1.06) | 15.82 (6.92) | 0.93 (1.11) |

*Table S6.* Bonferroni-corrected correlation analyses of difference scores (Drug minus placebo) of the STAI-state, baseline-corrected peak scores on the VAS, and emotional Stroop outcome variables with difference values of THC, 11-OH-THC, CBD, and 7-OH-CBD blood plasma concentrations (Drug minus placebo).

|  | **THC** | **11-OH-THC** | **CBD** | **7-OH-CBD** |
| --- | --- | --- | --- | --- |
| **STAI-state** |  |  |  |  |
| THC | ***τ*_b_(22)= 0.37, *p*= 0.05** | ***r*(22)= 0.66, *p*< 0.01** | *τ*_b_(22)= -0.03, *p*> 0.99 | N/A |
| THC/CBD | *τ*_b_(17)= 0.16, *p*> 0.99 | *τ*_b_(17)= 0.20, *p=* 0.83 | *τ*_b_(17)= 0.16, *p*> 0.99 | N/A |
| CBD | *τ*_b_(22)= 0.34, *p*= 0.10 | *τ*_b_(22)= 0.25, *p*= 0.41 | *τ*_b_(22)= 0.30, *p*= 0.18 | N/A |
| **VAS peak levels** |  |  |  |  |
| THC | *τ*_b_(20)= 0.27, *p=* 0.28 | *τ*_b_(20)= 0.23, *p*> 0.49 | *τ*_b_(20)= -0.286, *p*= 0.42 | N/A |
| THC/CBD | *τ*_b_(18)= 0.05, *p*> 0.99 | *τ*_b_(18)= 0.10, *p*> 0.99 | *τ*_b_(18)= 0.09, *p*> 0.99 | N/A |
| CBD | *τ*_b_(19)= -0.035, *p*> 0.99 | *τ*_b_(19)= -0.11, *p*> 0.99 | *τ*_b_(19)= 0.37, *p*= 0.09 | N/A |
| **Emotional Stroop** |  |  |  |  |
| **Number Correct** |  |  |  |  |
| THC | *τ*_b_(21)= -0.15, *p*> 0.99 | *r*(21)= -0.21, *p*> 0.99 | *τ*_b_(21)= 0.17, *p*> 0.99 | N/A |
| THC/CBD | *r*(17)= 0.27, *p*= 0.89 | *τ*_b_(17)= -0.06, *p*> 0.99 | *r*(17)= 0.18, *p*> 0.993 | N/A |
| CBD | *τ*_b_(21)= -0.07, *p*> 0.99 | *τ*_b_(21)= 0.25, *p*= 0.47 | *r*(21)= 0.20, *p>* 0.99 | N/A |
| **Reaction Time** |  |  |  |  |
| THC | *τ*_b_(21)= 0.12, *p*> 0.99 | *τ*_b_(21)= 0.09, *p*> 0.99 | *τ*_b_(21)= -0.11, *p*> 0.99 | N/A |
| THC/CBD | *r*(17)= -0.13, *p*> 0.99 | *τ*_b_(17)= -0.17, *p*> 0.99 | *r*(17)= -0.01, *p*> 0.99 | N/A |
| CBD | *τ*_b_(21)= -0.14, *p*> 0.99 | *τ*_b_(21)= -0.08, *p*> 0.99 | *τ*_b_(21)= -0.26, *p*= 0.31 | N/A |

*Note:* STAI: State-Trait Anxiety Inventory; VAS: Visual Analogue Scale; *τ*_b_: Kendall Tau correlation coefficient, *r:* Pearson correlation coefficient.
